# Enhancing bladder cancer awareness and knowledge through multifaceted community engagement in Egypt: a cross-sectional study

**DOI:** 10.3332/ecancer.2025.1886

**Published:** 2025-04-10

**Authors:** Ahmed A Hammad, Osama Ezzat, Alex Filicevas, Sara A Hammad, Mostafa M Elkady, Mohamed E Eladl, Karim Mosaad, Mohammed M Shalaby, Ahmed A Barakat, Karim Y El-Deeb, Basma A Eldawody, Ghada Elkanishy, Muhammed A Moukhtar Hammad, Ahmed Mosbah

**Affiliations:** 1 Faculty of Medicine, Mansoura University, Mansoura 35516, Egypt; 2 Urology Department, Urology and Nephrology Center, Mansoura University Faculty of Medicine, Mansoura 35516, Egypt; 3 World Bladder Cancer Patient Coalition, Brussels, Belgium

**Keywords:** bladder cancer, knowledge, community engagement

## Abstract

**Background:**

Bladder cancer (BC) poses a significant health challenge in Egypt, constituting 16% of male cancers and resulting in over 7,900 deaths annually. Globally, BC stands as the tenth most prevalent cancer with an anticipated 72.8% rise in new cases by 2040. As BC is an important health problem, early detection and prevention are crucial for improving outcomes. This could be done by increasing the disease’s awareness through structured campaigns for the community, increasing their insights about the risk factors of the disease; most commonly, smoking as well as other risk factors as exposure to certain chemicals, alcohol consumption, diet, obesity and schistosomiasis. The awareness includes not only the risk factors but also the most common early symptoms for detection.

**Objective:**

To compare the effect of our awareness campaign about BC in Mansoura, Egypt on the index of awareness measured by our locality-generated simplified questionnaire.

**Methods:**

The campaign featured symposiums, awareness booths and online initiatives that gathered substantial participation. We administered a purpose-designed survey (in either Arabic or English) comprising questions regarding the following: perception of BC as common cancer, BC’s risk factors acknowledgement, knowledge about screening and diagnosis, perception of the methods of treatment and the importance of screening. We used face-to-face surveys targeting the public in Mansoura to compare between the index of awareness about BC pre-awareness and post-awareness. In addition, online surveys targeting Egyptian medical students and medical staff were conducted to evaluate their knowledge about BC.

**Results:**

This study was conducted on 1,673 people (*n* = 304 in-person surveys and *n* = 1,369 online surveys). Of the 1,673 participants, 52.45% were females; the age of the responders ranged from 18 to 40 years. The mean index of awareness before our awareness in the campaign in the face-to-face phase was 58.52% while the index of awareness after our campaign increased to 94.28%.

**Conclusion:**

The public’s post-awareness survey responses revealed an enhancement in awareness of BC and highlighted the campaign’s effectiveness in disseminating critical information. Prospective long-term research is proposed to assess such awareness campaigns’ impact on patient survival and community burden.

## Background

Cancer is a group of diseases characterised by the uncontrolled growth and spread of abnormal cells that can result in death if not treated. Although the cause of most cancers is not well understood, numerous factors are known to increase risk, including many that are potentially modifiable (e.g., tobacco use and excess body weight) and others that are not (e.g., inherited genetic mutations) [1]. These risk factors may act simultaneously or in sequence to initiate and/or promote cancer growth [1]. Among the various types of cancer, bladder cancer (BC) is one of the most common cancers in Egypt representing about 16% of all cancers among men and accounting for more than 7,900 deaths per year [2]. Globally, BC is the tenth most common cancer with an approximate incidence of 573,000 new cases in 2020 [3]. About 213,000 people died of BC in 2020, with this disease ranking as the 13th most common cause of cancer-related death (and ninth among men) [3]. By 2040, the number of new BC cases is expected to increase by 72.8% to reach 991,000 patients diagnosed worldwide [4]. Moreover, the number of BC deaths was estimated to increase by approximately 86.6%, from 213,000 in 2020 to 397,000 in 2040 [4].

BC significantly impacts patients, families and the society. Besides the psycho-social burden, BC also places a huge economic burden represented in the health care system costs and consumption of treatment resources [5]. One study estimated that BC cost European Union healthcare systems about €2.9 billion in 2012, representing 59% of the total economic burden of the disease [6]. Inpatient care was the major cost component, accounting for 58% (€1.7 billion) of healthcare costs, followed by expenditures on medicines at €568 million (20% of total healthcare costs) [6]. 

Quality of life (QoL) is а multidimensional construct that encompasses physical, social, emotional and functional well-being. Health-related QΟL is а state of well-being with two components: (1) the ability to perform the tasks of daily living that reflect physical, psychological and social well-being, and (2) the individual’s satisfaction with levels of functioning and control of the disease and/or the associated treatment-related sequelae [7, 8]. In the context of cancer, an individual’s baseline functioning in these areas is impacted by the disease and its associated treatment. Cancer and its treatments, such as chemotherapy and radiation, often lead to a significant symptom burden, including pain, fatigue, changes in body image and diminished physical function. This can greatly impact patients’ QoL and overall well-being [9]. Additionally, a diagnosis of cancer is often associated with increased rates of depression and anxiety. These mental health challenges can exacerbate the perception of pain and reduce overall QoL [10]. Moreover, patients might experience social isolation due to their illness or the side effects of treatment, impacting their relationships and social interactions [11]. Besides, the financial burden of cancer treatment can significantly impact patients’ QoL and lead to significant economic stress. Financial toxicity includes direct costs like medical expenses and indirect costs like lost income [12].

The single most dangerous risk factor for developing BC is tobacco smoking [13]. With the increased prevalence of the disease in smoking populations, it is attributed in some reports to more than half of the cases [14]. Beta-naphthylamine and polycyclic aromatic hydrocarbons, which are carcinogens in tobacco smoke, increase inflammation and their metabolism ends in DNA adduct formation and irreversible genetic mutation. Other risk factors include occupational exposure to chemicals and arsenic contamination in drinking water. Past radiation exposure, chronic bladder inflammation and genetic predisposition can also increase the risk of BC. Alcohol consumption, high red meat intake and obesity are also considered risk factors for BC. Schistosomiasis infection, an endemic in the Middle East and Africa is famously associated with squamous cell carcinoma of the bladder [15].

Furthermore, the most common earliest presentation of BC is macroscopic haematuria [16]. World Bladder Cancer Patient Coalition Global BC Patient and Carer survey results show that visible blood in urine or macro-haematuria, was the most commonly experienced symptom by patients with BC [17]. Other presentations can be nonspecific lower urinary tract symptoms such as dysuria, frequency and urgency [16]. Physiological excretion of red blood cells is known to occur at low levels in healthy individuals [18]. Haematuria may result from trauma during sexual intercourse or urine may be contaminated during menstruation [19]. Vigorous exercise is also known to cause haematuria; therefore, this is a diagnosis of exclusion [19]. Pathological causes of haematuria can occur anywhere in the renal tract, from the glomerulus to the distal urethra [20]. Thus, great efforts should be directed toward public understanding, prevention and early disease detection by mass awareness that facilitates screening and timely diagnosis. Screening aims to identify disease at an earlier stage and improve morbidity and survival [21]. Screening for BC has been the subject of several previous studies. Britton *et al* [22] enrolled 2,356 men aged between ages 60 and 85, who were asked to perform home haematuria dipstick testing weekly for 10 weeks [22]. BC was diagnosed in 3.5% of the men, and no patient was diagnosed with non-papillary muscle-invasive bladder cancer (MIBC). At 7-year follow-up, none of the patients with low-grade papillary non-muscle invasive bladder cancer had died from BC or progressed to MIBC [23]. Earlier detection could, therefore, reduce muscle-invasion and metastasis and improve survival [23]. However, the most effective preventive strategy is to avoid predisposing factors [24]. BC incidence can be reduced by eliminating exposure to carcinogens or, where elimination of hazardous substances is not possible, lowering occupational and environmental exposure [25].

To date, and to the best of our knowledge, no Egyptian study has specifically addressed public awareness and perceptions of BC. While surveying individuals who have already received BC awareness education has its benefits, it is equally important to understand the perspectives of those who have not yet been exposed to such information. Given that almost everyone could benefit from awareness of BC and its risk factors at some stage in their life, particularly those at risk (individuals with predisposing conditions and their family members), it is essential to prospectively assess the general population’s awareness of BC, including their perceptions of symptoms and risk factors. Currently, there is a significant gap in the literature regarding this aspect. Therefore, with the ultimate goal of generating data to inform efforts aimed at ensuring prospective patients have accurate expectations of BC symptoms and risk factors, thereby encouraging early detection, we undertook this study.

Our objectives were to survey a sample of the Egyptian general population regarding their awareness of BC; assess their understanding of BC symptoms, risk factors, treatment options and screening methods; and evaluate their perceptions of the importance of early detection and prevention strategies. By collecting this data, we aim to create a baseline that will not only help in evaluating the impact of future public awareness campaigns but also assist in tailoring these campaigns to address specific knowledge gaps within the population in Egypt.

## Methodology

Enhancing BC awareness and knowledge in Egypt requires a multifaceted approach that involves community engagement, collaboration with healthcare professionals and empowering patients. We designed a plan to cater to two broad goals: empowering patient support groups and constructing a simplified awareness algorithm.

In our BC awareness campaign in Egypt, we have utilised the Health Belief Model (HBM) to guide our strategies. HBM is a theoretical model that can be used to guide health promotion and disease prevention programs [26]. It is used to explain and predict individual changes in health behaviors. It is one of the most widely used models for understanding health behaviors. Key elements of the HBM focus on individual beliefs about health conditions, which predict individual health-related behaviors. The model defines the key factors that influence health behaviors as an individual’s perceived threat to sickness or disease (perceived susceptibility), belief of consequence (perceived severity), potential positive benefits of action (perceived benefits), perceived barriers to action, exposure to factors that prompt action (cues to action) and confidence in ability to succeed (self-efficacy).

We applied the key components of the HBM in our campaign by focusing on perceived susceptibility, perceived severity, perceived benefits, perceived barriers, cues of action and self-efficacy. Perceived susceptibility is how individuals evaluate their chance of getting a certain illness. Our campaign objective was to raise awareness about the risk factors for BC, including smoking, occupational hazards and schistosomiasis, which is a common disease in Egypt. To educate the public about the dangers of BC, we organised workshops and handed out brochures, stressing that anyone, particularly those with these risk factors, could be at risk. As for perceived severity, it is an individual’s understanding of how dangerous they think they are at getting the disease and what might happen to them if they do get it. We emphasised the severe health consequences of BC, such as its effect on daily life and the risk of death if not diagnosed and treated promptly. We employed presentations by medical professionals and statistical data to illustrate the serious outcomes of untreated BC, to instill a sense of urgency and significance regarding early detection and treatment.

Regarding perceived benefits, we highlighted the advantages of routine check-ups and identifying BC at an early stage. We clarified that finding a problem sooner can result in more successful treatment and improved results. Although we couldn’t find specific success stories or testimonials from participants due to their reluctance, we concentrated on general evidence and data from healthcare studies to demonstrate the effectiveness of early screening. The individual’s assessment of the challenges to changing their behavior is what we call perceived barriers. Some of the obstacles that people in Egypt face when dealing with cancer are not being able to get adequate medical help, following traditions that may not be helpful and feeling ashamed or embarrassed about having cancer. We have also incorporated various cues to action in our campaign, such as educational sessions, media campaigns and reminders from healthcare providers. We used posters, and social media posts, to remind individuals about the importance of getting checked for BC. Community health workers also played a crucial role in delivering these cues directly to the people. To enhance self-efficacy, we provided educational programs that offered information and empowered individuals with the skills and confidence to seek medical advice and undergo screenings. In summary, HBM has been instrumental in structuring our BC awareness campaign in Egypt. By understanding and addressing the beliefs and attitudes of our target population, we have designed interventions that effectively promote BC awareness and encourage early detection.

Patient support groups are an integral part of the cancer care ecosystem and serve as a valuable resource for patients, including information and educational resources, emotional support and navigation through the disease with guidance from people who have been affected by BC themselves. The Global BC Patient and Care Survey results show that 50% of respondents contacted a patient organisation and/or charity for support. Of those who did, 96% received the information and support they needed either fully or to some extent [27]. 

Before commencing the study, approval was sought from the ethics committee of the Faculty of Medicine at Mansoura University in Egypt. Participants were informed about the study’s purpose and assured of the confidentiality measures in place to protect their personal information and responses. Importantly, participants’ involvement was entirely voluntary, anonymous and based on their individual choice.

**Figure 1.**
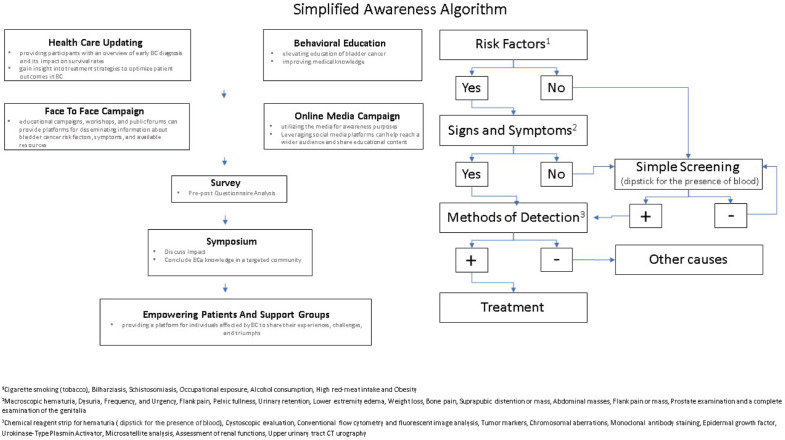
Simplified awareness Algorithm. Simplified constructed algorithm for BC awareness and early detection.

As illustrated in (Figure 1), public education is the cornerstone of the campaign, informing the target population of the alarming risk factors and possible existing symptoms/signs that arouse early detection and diagnosis of the disease. Besides face-to-face awareness, online media could be utilised to handle awareness symposiums, collect data and perform public questionnaires and mass surveys. All the mentioned tools would assist in empowering BC patients as well as support groups.

By implementing the strategies mentioned above, we were able to raise awareness, improve early detection rates and provide support to individuals affected by BC. This was the focus of our activities for the ‘BC Awareness Month 2023’ held by our Mansoura University Safety Society (MUSS) team throughout May 2023 in Mansoura, Egypt. This initiative was organised with the patronage of the experts and leading professors of nephrology and urology. Healthcare professionals’ engagement started through an online orientation session in collaboration with a consultant urologist. The awareness booth initiatives were held in separate locations, including a public sporting club on 19th May, Mansoura University Specialised Hospital on 21st May, and outpatient clinics in Mansoura hospitals on 22nd May–23rd. On 24th May at Mansoura Faculty of Medicine, MUSS held a symposium to raise awareness about BC.

Throughout those events, we conducted a two-part study. In the first part of the study, a self-constructed questionnaire was addressed to the public, using our own knowledge index to assess our campaign by comparing pre-test responses to post-test responses. The questionnaires were provided in Arabic, the official language of Egypt and in English for those who preferred it. To ensure consistency between the two versions, a bilingual consultant urologist translated the original survey to Arabic and back translated it to English. The questionnaire was piloted using a convenience sample of 17 individuals. We developed a survey on the basis of assessing the awareness of the general public regarding the subject of BC.

The survey started with acquiring personal and demographic information of each applicant regarding their name, age, sex and occupation. We included questions targeting the individual’s general knowledge of the disease by asking if he or she has ever heard about BC, thinks BC is prevalent, and can approximate the number of individuals affected yearly. We also assessed the knowledge of the applicant regarding the sign and symptoms, emphasising the most alarming ones like haematuria, fever and weight loss. In addition to that, we highlighted risk factors that can put individuals at higher risk including radiation, occupational hazards and smoking. We asked about common misconceptions, such as if the disease can be inherited and if the disease affects only males. Further questions were about the possible utilities used to diagnose BC, how BC is treated, and if there is a possibility of recurrence after treatment. We also assessed individual’s beliefs about the psycho-social aspect of the patients suffering from BC by providing questions on whether BC patients suffer from any stigma and whether the applicants agree or not with such stigma. Finally, we evaluated how likely the applicant is to undergo screening for BC by urinalysis and how important the idea of screening is for him or her. Feedback received was taken into consideration to improve the clarity of the questionnaire. Pre- and post-surveys were administered by a team of 26 bilingual medical students to the public at a local park booth for assessment of participants’ knowledge before and after receiving an extensive orientation on BC.

## Results

For the first part of the study, 304 responses were collected. Participants were randomly approached by individuals, of both sexes, at a public club. They were eligible to participate if they were 18 years of age or more and appeared to be in a good mental health state. Participants had the choice of answering the questionnaires on paper or digitally. Participants who did not wish to continue at any point in either of the questionnaires or the orientation were not included in the study. The orientation was introduced, utilising a standardised fact sheet developed with guidance from the BC Advocacy Network [27]. The analysis focused on the laypersons’ understanding of BC’s severity, frequency, signs, symptoms, associated risk factors and screening tests. Participants who were interested in knowing more about BC were provided with further resources to look up the topic. The demographic breakdown revealed that 51.3% of participants were aged between 20 and 40 years. Gender distribution was relatively balanced, with 51.8% being male. Regarding education, nearly half of the participants (49.3%) held a college degree.

**Figure 2.**
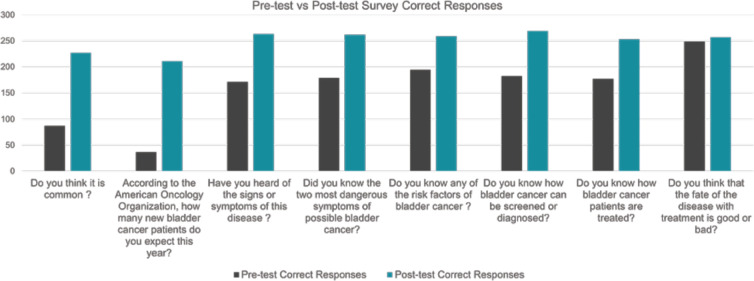
BC awareness month, 2023: results of awareness campaign to general public survey results. Correct public responses to assess the awareness campaign by comparing pre-test responses to post-test responses.

As illustrated in Figure 2, the perception of BC as a common cancer, pre-awareness results showed that only 29% of participants thought BC was common. After the awareness intervention, this perception significantly increased to 84.7%, indicating a substantial rise in awareness. In regard to the perception of the methods of treatment, before the intervention, 59.4% of participants knew how BC patients are treated. This figure rose dramatically to 94.1% afterward, showing a significant increase in treatment awareness. Concerning risk factors acknowledgment, it also saw a notable improvement. Initially, 65.1% of participants were aware of the risk factors, which increased to 95.9% after the intervention. Awareness of how BC can be tested for or diagnosed was initially at 60.8%. This awareness reached 100% post-intervention, highlighting a complete success in this educational aspect. Apropos of the Importance of Routine Screening, 78.3% of participants thought it was important to get routinely screened for BC before the intervention. Afterward, this belief increased to 96.7%, reflecting a significant enhancement in the understanding of routine screening’s importance.

Regarding the second part of the study, the participants were Mansoura University Faculty of Medicine affiliates. We used an online version of the questionnaire that we utilised in the first part of the study, receiving 1,369 responses, to evaluate their recognition of BC disease. Along with attending the campaign, all faculty members and students were eligible to take part in this online questionnaire. Here, 67.1% of the participants were aged between 18 and 20 years, indicating a younger demographic. The majority were female, comprising 56.7% of the participants. A significant portion, 79.9%, had a college degree, showing a highly educated group.

**Figure 3.**
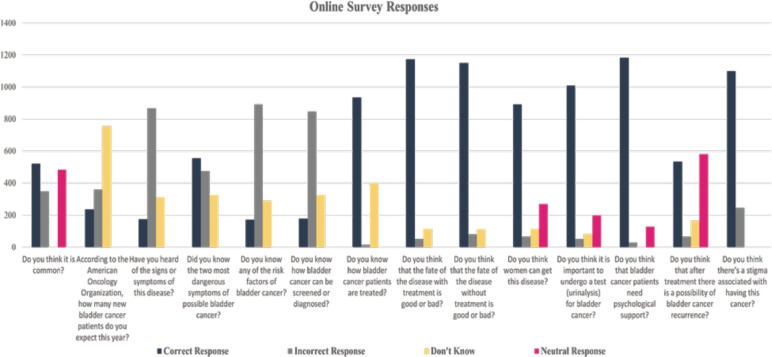
BC knowledge in an underserved, highly afflicted population online survey results. Responses of questions that assess the recognition of BC disease among Mansoura University Faculty of Medicine affiliates.

As to the perception of BC as a common cancer in this group, 38.6% of participants initially thought BC was common, as shown in Figure 3. Regarding the perception of the methods of treatment, a substantial 85.6% acknowledged that without treatment, BC has a bad prognosis. Additionally, 87.4% knew that with treatment, it has a good prognosis. Furthermore, 69.3% could identify ways of treatment, showcasing a relatively high initial awareness. Awareness of the most dangerous risk factors was at 40.5% and 50.4% knew that tobacco smoking is the most important risk factor for BC. This indicates a moderate level of awareness among participants. As for knowledge about screening and diagnosis, only 13.2% of participants knew how BC is screened or diagnosed, indicating a significant gap in awareness in this area. As regards to the importance of routine screening, a majority, 75.1%, believed that urinalysis screening for BC is crucial for the public, reflecting a strong initial understanding of the importance of routine screening.

## Discussion

BC is one of the most common cancers, and its incidence is only expected to increase. The study was created to evaluate public information about BC by several awareness tools. The primary objective of this project was to raise awareness within the community. This was accomplished through community education about the most pertinent risk factors prevalent in Egypt and the early warning signs of the disease. During all interactions with individuals, whether in public areas or outpatient clinics, we provided information about the disease and preventive measures. Additionally, we distributed brochures containing comprehensive information on all risk factors, symptoms and signs, with a specific focus on tobacco smoking as a risk factor and macroscopic hematuria as a sign. By raising awareness, individuals experiencing these symptoms will be encouraged to undergo screening, enabling early detection and prevention of the disease from progressing to advanced stages.

The results of our study demonstrated that the participants had little knowledge about BC before the start of the awareness campaign. Most people did not think that BC was a common type of cancer. Moreover, the understanding of the risk factors for BC was insufficient, with under two-thirds of the participants being aware of them before the campaign started. Furthermore, many participants did not know about the screening and diagnostic options for BC. It is worth noting that a few participants were aware that women could also have BC. The participants had limited knowledge about the available treatments and the likely outcomes, with only half of them being aware of this information. These results emphasise the urgent need for focused educational initiatives to enhance public awareness and comprehension of BC.

It is important to note the success of our BC awareness campaign, and it can be attributed to several key factors. First, by strategically organising the campaign in public areas such as clubs and outpatient clinics, we ensured maximum visibility and accessibility to a diverse audience. Second, our emphasis on providing educational content and raising awareness about BC resonated with participants’ desire for knowledge, motivating them to engage with the campaign. Moreover, by incorporating various activities for both children and adults in the club setting, we created an inviting atmosphere that appealed to families and individuals alike. Activities such as interactive games and informative sessions captivated the audience’s interest, fostering an environment conducive to learning and participation. Finally, the allure of a giveaway at the conclusion of the campaign incentivised individuals to actively participate, as they eagerly sought the opportunity to win prizes. This combination of factors – strategic location, educational content, engaging activities and enticing incentives – played a pivotal role in encouraging widespread participation and engagement with our BC awareness campaign.

However, it is noteworthy to point out several limiting factors in our study. We cannot extrapolate from our findings to draw firm conclusions regarding awareness and perceptions of BC among the whole Egyptian population; however, our data suggest that there is room to improve the public understanding of BC in Egypt, and there is much to potentially be gained by such improvements. In addition, we utilised a self-constructed survey to evaluate public knowledge. Since there are not any prior studies in Egypt regarding awareness of BC, we have only used the BC Advocacy Network awareness material as a guide for constructing the survey that tailors to a very generic covering of the topic.

## Conclusion

This study suggests the lack of knowledge among an educated sector of the Egyptian public and medical students’ population toward BC early signs, diagnostic tests and risk factors including its association with tobacco smoking. Despite that, through the awareness campaign, participants were able to acknowledge the most important facts about BC.

Public education should be intensified towards cessation of smoking and early detection of the disease by rapid urological consultation in cases of hematuria. We should benefit from all available tools including online media to enhance awareness and understanding of BC. International BC awareness campaigns can help further strengthen the awareness through multi-country collaboration and especially with local adaptations of resources and materials. Further long-term research is needed to investigate the outcome of these awareness campaigns on patient survival and community burden.

We should support such underserved communities with more awareness campaigns, including and not limited to mental and psychological support. Effective prevention of BC necessitates the dissemination of clear information and prevention messages to the general population.

## Conflicts of interest

Alex Filicevas is an employee of the World Bladder Cancer Patient Coalition, which receives funding from Astellas, AstraZeneca, Bristol Myers Squibb, Ferring, Janssen, Merck, MSD, Pfizer, Roche and Seagen. Alex Filicevas is a volunteer board member of All. Can International, which receives funding from Amgen, Bristol Myers Squibb, Johnson and Johnson, Illumina and Roche. All other authors have no conflicts of interest.

## Funding

No funding.

## Informed consent

Not applicable.

## Author contributions

**Table:** 

AAH:	Data collector and Paper editorial	Took the lead in writing the manuscript with support from OE. Performed the analytic calculations and carried out paper studies Strategies of bladder cancer awareness Contributed to the final version of the manuscript
OE:	Data collector and Paper editorial	Reviewed and supervised paper Helped shape the research Contributed to the final version of the manuscript
AF:	Data collector and Paper editorial	Empowering patients and support groups Helped shape the research Contributed to the final version of the manuscript
SAH:	Data collector	A brief overview of bladder cancer aetiology and symptoms. Carried out paper studies.
MME:	Data collector	A comprehensive overview of bladder cancer screening and prevention outcomes.
MEE:	Data collector	A brief breakdown of bladder cancer risk factors and its most common clinical presentations.
KM:	Data collector	The role of a number of tools that can be used in prevention and early detection of bladder cancer, such as behavioral education, health care updating, and screening of high-risk patients.
MMS:	Data collector	An algorithmic approach for awareness and enhancement of bladder cancer early detection.
AAB:	Data collector	The role of pushing for more community education on bladder cancer and the significance of hematuria.
KYE: BAE:	Data collector Data collector	Prevalence and mortality of bladder cancer worldwide and in Egypt, and the expected incidence by 2040. A brief overview on key content areas, research purpose, relevance of the work, and main outcomes
MAH:	Paper editorial	Helped shape the research and analysis Carried out studies Contributed to the final version of the manuscript
GE:	Supervisor and director	Supervised the project
AM:	Supervisor and director	Supervised the project
